# Transcriptional profiling by cDNA-AFLP analysis showed differential transcript abundance in response to water stress in *Populus hopeiensis*

**DOI:** 10.1186/1471-2164-13-286

**Published:** 2012-06-29

**Authors:** Yuepeng Song, Zeliang Wang, Wenhao Bo, Yuanyuan Ren, Zhiyi Zhang, Deqiang Zhang

**Affiliations:** 1 National Engineering Laboratory for Tree Breeding, College of Biological Sciences and Technology, Beijing Forestry University, Beijing, 100083, P. R. China; 2 Key Laboratory of Genetics and Breeding in Forest Trees and Ornamental Plants, Ministry of Education, College of Biological Sciences and Technology, Beijing Forestry University, Beijing, 100083, P. R. China

## Abstract

**Background:**

Drought is one of the main environmental factors limiting tree growth and productivity of plantation forests worldwide. *Populus hopeiensis* Hu et Chow is one of the most important commercial plantation tree species in China. However, the genes controlling drought tolerance in this species have not been identified or characterized. Here, we conducted differential expression analyses and identified a number of genes that were up- or downregulated in *P*. *hopeiensis* during water stress. To the best of our knowledge, this is the first comprehensive study of differentially expressed genes in water-stressed *P*. *hopeiensis*.

**Results:**

Using the cDNA-AFLP detection technique, we used 256 primer combinations to identify differentially expressed genes in *P*. *hopeiensis* during water stress. In total, 415 transcript derived-fragments (TDFs) were obtained from 10× deep sequencing of 473 selected TDFs. Of the 415 TDFs, 412 were annotated by BLAST searches against various databases. The majority of these genes encoded products involved in ion transport and compartmentalization, cell division, metabolism, and protein synthesis. The TDFs were clustered into 12 groups on the basis of their expression patterns. Of the 415 reliable TDFs, the sequences of 35 were homologous to genes that play roles in short or long-term resistance to drought stress. Some genes were further selected for validation of cDNA-AFLP expression patterns using real-time PCR analyses. The results confirmed the expression patterns that were detected using the cDNA-AFLP technique.

**Conclusion:**

The cDNA-AFLP technique is an effective and powerful tool for identifying candidate genes that are differentially expressed under water stress. We demonstrated that 415 TDFs were differentially expressed in water-stressed poplar. The products of these genes are involved in various biological processes in the drought response of poplar. The results of this study will aid in the identification of candidate genes of future experiments aimed at understanding this response of poplar.

## Background

Drought stress is one of the main environmental constraints that severely affects plant growth and development [1]. Worldwide, water stress is the main cause of economic losses in agriculture and forestry. Water is an increasingly scarce resource given the current and future needs of human populations and societies [2], and droughts are expected to become increasingly severe due to climate change and the increasing scarcity of water. Perennial plants have evolved specific physiological mechanisms to adapt to natural variation in environmental conditions, including variation in water availability [3]. Plants under drought stress may survive by employing various protective mechanisms [4]. Analysis of these protective mechanisms will contribute to our knowledge of drought-tolerance and drought-resistance [5].

Currently, molecular biologists are exploring the genetic basis of plant responses to environmental stresses, including drought, by examining the expressions of relevant genes [6]. Drought tolerance is a complex trait that is regulated by multiple genes, interactions among genes, and environmental cues [7]. Therefore, manipulating a single gene encoding a protein or transcription factor has proved to be insufficient to maintain productivity under drought conditions [8]. Hence, the complex responses to environmental stress, from perception to transcriptional changes, need to be considered on a systems biology scale [5]. Several techniques are available for such studies, including representational difference analysis (RDA), serial analysis of gene expression (SAGE), suppression subtractive hybridization (SSH), cDNA microarrays, oligo microarrays, and RNA-Seq to analyze the transcriptome and identify genes that are up- or downregulated during drought tolerance [9-11]. The cDNA-AFLP method is an extremely efficient and sensitive mRNA fingerprinting technique to identify both common and rare transcripts [12,13]. This technique is a robust and high-throughput tool for analysis of genome-wide gene expression, and can be used for identifying genes that are differentially expressed under abiotic stress [14].

Poplar has become a model species for tree research since the release of the full *P*. *trichocarpa* genome [15]. In addition, poplars are the most rapidly growing trees at temperate latitudes, and are widely grown for pulp, paper, wood, and fuel [16]. Poplar is more sensitive to water deficit than other tree species; however, its drought tolerance varies markedly among different *Populus* genotypes, both inter-and intra-specifically [17,18]. Many recent genome-wide studies have provided insight into the variability of the transcriptome response to water stress. Street et al. (2006) hypothesized that the control of gene expression may be an important process in species divergence [19]. Wilkins et al. (2009) suggested that it is difficult to capture a genome-wide drought response using only one or a few *Populus* genotypes [20]. Moreover, differences in the drought-responsive transcriptomes among balsam poplar genotypes are related to the extent of intra-specific DNA sequence variation [21]. These results suggest that genetic relatedness is likely an indicator of a shared drought response [22]. Considering the variability in drought transcriptomes of various forest trees, the use of more drought-tolerant *Populus* genotypes used in research would help to elucidate the drought response of poplar.

*Populus hopeiensis*, which is in the section *Leuce*, is native to China. It is a drought- and chill-tolerant tree species that is mainly distributed in the arid and semi-arid areas of northwest and northern China (Additional file 1: Figure S1). Therefore it is an ideal material for the study the mechanisms of drought-tolerance of mechanisms in poplar. The purpose of the present study was to analyze the transcriptome response to water deficit, and to highlight candidate genes for future research.

## Results

### Sequence analysis of cDNA clones

**Figure 1:**
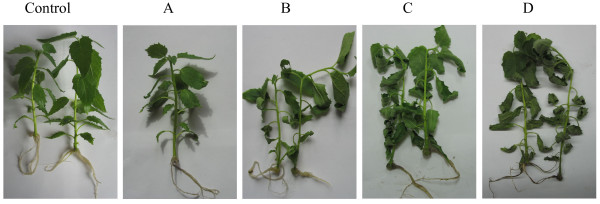
**Water-stressed *P* . *hopeiensis* plantlets.** Control: No water-deficit treatment. **A**–**D**, 10%, 20%, 30%, and 50% fresh weight loss treatment.

**Figure 2:**
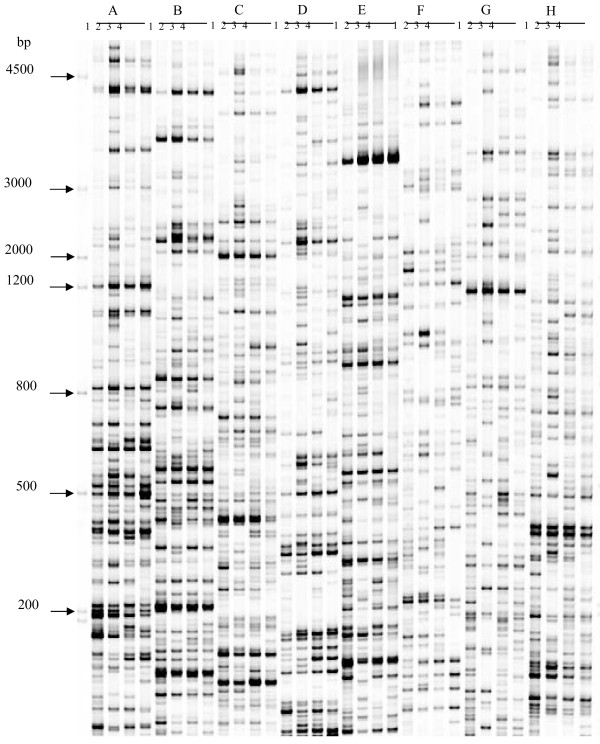
**Polyacrylamide gels of *P.*  *hopeiensis* transcript profiles at different water deficit treatment stages.** Marker molecular weights are indicated on the left. 1, 2, 3, and 4 correspond to fresh weight loss 0%, 20%, 30%, and 50%, respectively; **A**–**H**, Different primer combinations.

A total of 256 primer combinations (Additional file 2: Table S1) were used for the AFLP analysis of cDNAs from drought-stressed poplar. cDNAs were obtained from poplar at four stages, i.e. 0%, 20%, 30%, and 50%, of fresh weight loss (FWL) (Figures 1, 2). A total of 415 transcript-derived fragments (TDFs) was isolated from the silver-stained cDNA-AFLP gels based on their presence/absence (qualitative varints) or difference in the levels of expression (quantitative variants). A majority of the variants was of quantitative type (86%), whereas 14% were qualitative. Sequence alignment of these 415 cloned TDFs against NCBI databases showed that 412 had a homologous gene in the database. Due to incompleteness of poplar gene annotation and the conservation of gene families between poplar and *Arabidopsis*, we used the *Arabidopsis* matches of the identified differentially expressed genes as surrogates for function annotation. A total of 24 of 412 TDFs had no annotation in the database. Three of the TDFs (TDFs45-1, TDF183, and TDF280-1) had no similar sequences in the NCBI databases.

Among the TDF sequences, some high-homology sequences were from the same gene, for example, TDF214 and TDF493 were from the *DEHYDRIN* (*DHN*) gene: TDF358 and TDF421 were from the *BOILING STABLE PROTEIN* (*BSP*) gene, TDF221 and TDF377 were from a zinc-finger protein gene: TDF18, TDF80, and TDF91 were from an alpha expansin gene; and TDF25 and TDF157 were from a translation extension factor gene. TDF186, TDF313, TDF363, and TDF488 all represented members of the *PHOSPHATASE 2 C* gene family.

### TDF functional classification and expression analysis

**Figure 3:**
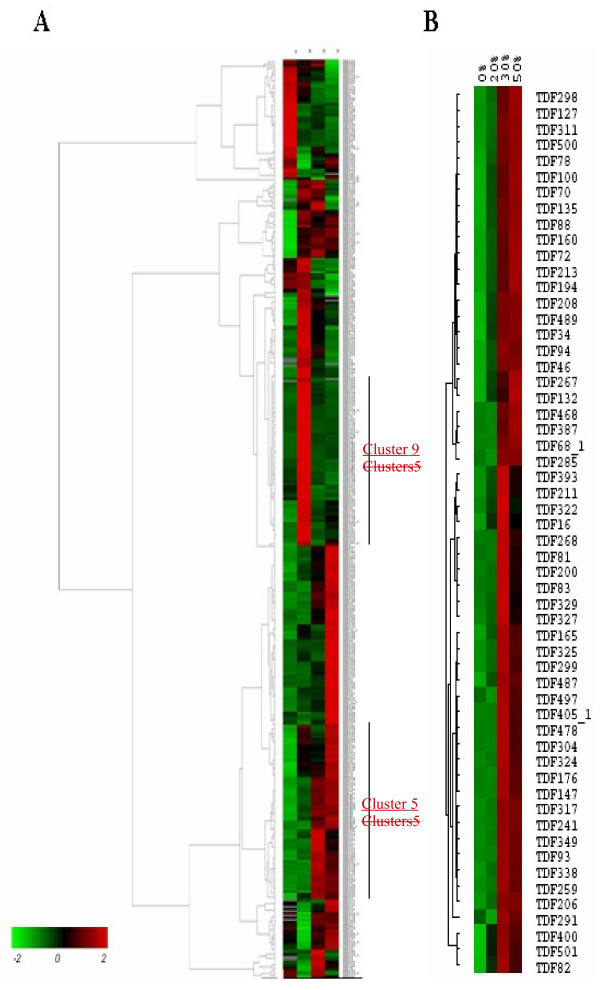
**Pearson’s correlation coefficient (PCC) heat map representing the relative differentially expressed *P* . *hopeiensis* transcripts during water deficit on the four harvest dates (minimum two-fold change).** Red indicates higher and green indicates lower levels of transcript. Data are row normalized. The 0%, 20%, 30%, and 50% correspond to the four harvest dates with increasing fresh weight loss. **A**, All transcript differential fragments (TDFs). **B**, Partial clusters of transcriptional changes.

**Figure 4:**
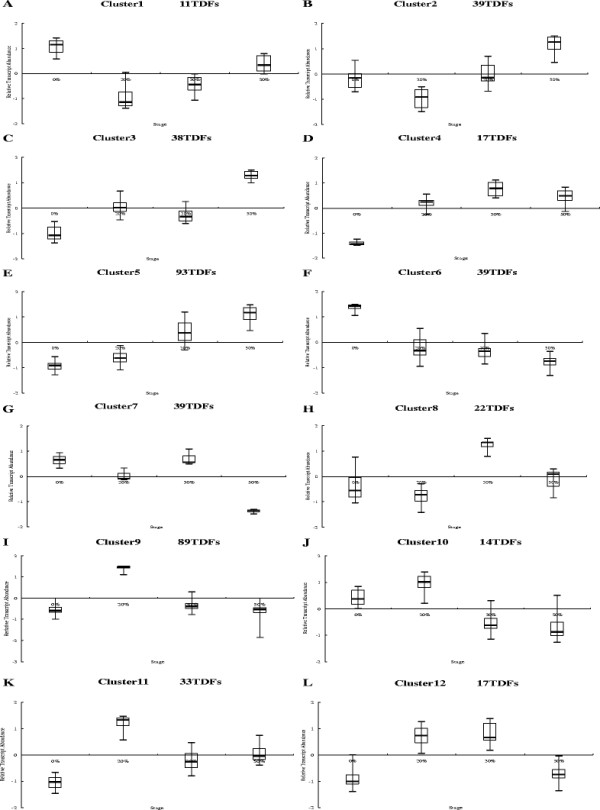
**Box plot illustrating the results of cluster analysis.** All transcript patterns of 415 TDFs were clustered into 12 groups. **A**–**L**, 12 clusters. Stage, the four water treatment time points. See text for the details.

To group genes with similar expression patterns, all TDFs were analyzed using hierarchical average linkage clustering. The 415 TDFs formed 12 clusters. Of these, 6 clusters of genes (Cluster 5, cluster 6, cluster 7, cluster 8, cluster 9, and cluster 10) were shown up regulated from 0% FWL to 20% FWL stage, the others down regulated. At 20% FWL to 30% FWL stage, 8 clusters of genes (Cluster 1, cluster 2, cluster 3, cluster 5, cluster 9, cluster 10, cluster 11 and cluster 12) were shown up regulated. At the last stage, only 5 clusters of genes (Cluster 3, cluster 4, cluster 8, cluster 9, and cluster 11) (Figures 3, 4). Clusters 5 and 9 contained 93 and 89 TDFs, respectively, and these two groups combined to form the largest cluster (43.8% total sequences). Genes in cluster 5 exhibited increased expression as the fresh weight decreased, peaking at the 50% FWL stage (Figure4). Genes in cluster 9 showed a peak in expression at the 20% FWL stage, which decreased expression at the 50% FWL stage. Clusters 5 and 9 contained TDF103, TDF108, TDF107, TDF128, TDF151, TDF186, TDF232, TDF363, TDF399, TDF404, TDF482 and TDF494, which encoded protein kinases, phosphokinases, transcription factors, heat shock protein (HSP), early-responsive to dehydration (ERDs), and late embryonic proteins(LEAs), respectively. The smallest cluster was cluster 7, which contained only three TDFs. The other eight clusters each contained 2–9% of the remaining TDFs.

### Gene ontology annotation

All TDF sequences were functionally and structurally annotated using Blast2GO, and the main assignments are summarized in Table1 under the three main categories of biological process, molecular function and cellular complement. Concerning the biological process ontology, metabolic process (GO: 0008152), localization (GO: 0051179), cellular process (GO: 0009987) and response to stimulus (GO: 0050896) were highly enriched for water loss 49.1% on average. The most enriched molecular function terms were binding (GO: 0005488), catalytic activity (GO: 0003824), structural molecule activity (GO: 0005198). Of those, enrichments, the percentages of the binding and catalytic activity terms were increased with water loss in upregulated volume, and decreased slowly in downregulated volume. In terms of cellular component ontology, the most significantly enriched terms was intracellular (GO: 0005622), which accounted for almost one-third of all terms. Similarly, membrane (GO: 0016020), nucleus (GO: 0005634) and apoplast (GO: 0048046) together comprised one third of cellular component terms. The most important GO terms of enrichment are indicated in Table1.

**Table 1:** GO categorization of differentially expressed poplar genes during water-deficit treatment

**Go category**	**Function category**	**20%FWL**		**30%FWL**		**50%FWL**	
		**UP(%)**	**Down (%)**	**UP (%)**	**Down (%)**	**UP (%)**	**Down(%)**
**Biological Process**	**Metabolic process**	27.9	21.1	24.5	28.2	27.7	28.9
	Oxidation reduction	7.0	6.1	7.2	7.4	8.4	7.1
	Carbohydrate metabolic process	4.6	-	4.8	3.3	4.8	3.4
	Regulation of transcription	3.2	4.3	-	4.1	4.3	3.2
	**Localization**	7.5	6.2	8.1	7.1	5.0	9.3
	Transport	3.8	5.1	4.3	6.1	4.7	5.0
	Intracellular protein transport	1.9	-	3.0	-	-	3.4
	**Cellular process**	7.6	12.2	7.9	13.5	6.2	11.1
	Protein amino acid dephosphorylation	4.5	-	-	-	-	-
	Protein amino acid phosphorylation	-	6.8	4.2	5.4	4.6	4.0
	**Response to stimulus**	2.4	1.1	4.7	1.6	2.9	2.5
**Molecular Function**	**Binding**	23.2	27.1	25.8	28.4	27.9	27.7
	Nucleotide binding	7.9	7.2	6.7	8.1	8.1	7.6
	ATP binding	5.4	8.0	7.1	7.2	7.4	7.2
	Protein binding	2.9	2.1	3.1	3.4	3.0	4.0
	Metal ion binding	4.1	3.2	4.1	3.3	6.2	4.1
	**Catalytic activity**	15.6	21.1	18.2	18.8	19.1	16.2
	Protein Kinase activity	-	3	2	3	-	3
	Protein serine/thromine kinase activity	-	3	-	-	-	2
	Hydrolase activity	4.5	-	3.2	3.6	3.2	3.8
	Nucleoside-triphosphatase activity	-	-	-	-	2	-
	Oxidoreductase activity	4.2	4.1	4.6	4.4	4.6	4.3
	Transferase activity	2.1	4.8	3.8	3.4	4.3	-
	**Structural molecule activity**	-	-	-	3.8	-	-
**Cell Complement**	**Intracellular**	43.3	31.5	30.1	40.4	35.5	27.9
	Chloroplast	-	5.5	2.8	-	-	-
	Cytoplasm	19.8	5.7	13.8	11.2	20.1	9.8
	Ribosome	9.8	5.2	4.4	10.1	4.6	7.7
	**Membrane**	24.8	28.1	18.2	20.2	14.4	22.2
	Extrisic to membrance	3.1	-	-	4.5	-	2.9
	Integral to membrane	11.0	8.9	12.3	13.1	10.8	13.3
	**Nucleus**	10.1	10.9	12.3	-	8.5	8.3
	**Apoplast**	3.6	-	-	4.3	-	3.9

“-”, the missing. Pie chart is shown in Additional file 3: Figure S2, Additional file 4: Figure S3, Additional file 5: Figure S4.

**Table 2:** **Transcript derived fragments (TDFs) from *P* . *hopeiensis* showing drought-responsive expression, with homologies to genes in the *P* . *trichocarpa* genome database**

**TDFs**	**Locus/Gene Model**	**Aliases**^**a**^	**Annotation**	**E-value**	**Accession no.**^**b**^	**ATG Locus/Gene Model**
TDF48	POPTR_0011s09860	---	NAD-dependent malate dehydrogenase	3.40E-50	JK475089	AT3G15020
TDF63	POPTR_0019s15110	Pt-CYP73.3	Trans-cinnamate 4-monooxygenase.	0	JK475090	AT2G30490
TDF70	POPTR_0001s16730	Pt-ALDH7.1	Aldehyde dehydrogenase	6.40E-85	JK475091	AT1G54100
TDF89	POPTR_0002s05660	Pt-OEE2.2	Similar to photosystem II oxygen-evolving complex protein 2	1.50E-122	JK475092	AT2G30790
TDF91	POPTR_0010s17440	PtEXPA3	Rare lipoprotein A (RlpA)-like double-psi beta-barre	3.80E-106	JK475093	AT2G03090
TDF98	POPTR_0016s00210	---	Sodium/hydrogen exchanger protein	1.00E-85	JK475094	AT1G79610
TDF125	POPTR_0001s01380	---	Dehydration-responsive protein	1.20E-80	JK475095	AT4G19120
TDF126	POPTR_0012s02430	---	rRNA 2'-O-methyltransferase fibrillarin	7.20E-128	JK475096	AT5G53060
TDF137	POPTR_0001s36870	---	Similar to early-responsive to dehydration stress protein (ERD4)	2.60E-96	JK475097	AT1G80780
TDF139	POPTR_0010s21710	---	Photosystem 2 reaction center PsbP family protein	0	JK475098	AT2G39470
TDF171	POPTR_0008s16110	Pt-IAA14.1	Auxin-responsive protein IAA	1.80E-96	JK475099	AT4G14550
TDF181	POPTR_0006s13650	---	Alanine dehydrogenase/PNT, N-terminal domain	6.20E-73	JK475100	AT4G33150
TDF189	POPTR_0016s09240	---	Isocitrate dehydrogenase (NAD+)	4.70E-99	JK475101	AT5G03290
TDF196	POPTR_0001s00550	---	Chromodomain-helicase-DNA-binding family protein	3.40E-131	JK475102	AT3G53030
TDF273	POPTR_0005s19710	Pt-ATR1.1	NADPH-ferrihemoprotein reductase	1.90E-52	JK475103	AT4G24520
TDF285	POPTR_0001s19100	---	Taurine catabolism dioxygenase TauD, TfdA family	6.90E-94	JK475104	AT3G21360
TDF287	POPTR_0003s13310	Pt-F.1	Ferric reductase like transmembrane component	9.90E-94	JK475105	AT1G64060
TDF295	POPTR_0001s04990	Pt-FAD2.2	Fatty acid desaturase	2.40E-179	JK475106	AT3G12120
TDF325	POPTR_0014s15710	Pt-ACO1.4	1-aminocyclopropane-1-carboxylate oxidase	6.10E-141	JK475107	AT1G12010
TDF333	POPTR_0013s12730	---	Pristanoyl-CoA/acyl-CoA oxidase	1.70E-67	JK475108	AT1G06310
TDF360	POPTR_0017s02330	---	NADH-ubiquinone oxidoreductase	1.10E-163	JK475109	AT5G37510
TDF361	POPTR_0011s11390	---	S)-2-hydroxy-acid oxidase	8.10E-44	JK475110	AT3G14415
TDF370	POPTR_0005s15660	---	bisphosphate carboxylase small chain 1A	4.40E-75	JK475111	AT1G67090
TDF372	POPTR_0003s13370	---	K + potassium transporter	4.60E-162	JK475112	AT4G23640
TDF382	POPTR_0012s09570	Pt-GAPDH1.1	Glyceraldehyde-3-phosphate dehydrogenase	1.20E-35	JK475113	AT3G04120
TDF388	POPTR_0002s05640	---	Aldo/keto reductase family protein	5.70E-84	JK475114	AT5G37570
TDF399	POPTR_0004s17980	Pt-ERD7.1	Early-responsive to dehydration 7	0	JK475115	AT2G17840
TDF405	POPTR_0003s08760	Pt-GDCH.3	Glycine cleavage system protein H precursor	9.30E-113	JK475116	AT2G35370
TDF417	POPTR_0002s00210	---	Mitochondrial carrier protein	7.70E-115	JK475117	AT5G26780
TDF418	POPTR_0006s10200	---	Iron/ascorbate family oxidoreductases	8.30E-136	JK475118	AT2G30830
TDF440	POPTR_0007s06400	Pt-IFS1.44	Cytochrome P450 CYP2 subfamily	2.10E-94	JK475090	AT4G34490
TDF459	POPTR_0002s08230	Pt-ALDH3.1	Aldehyde dehydrogenase	3.00E-68	JK475109	AT1G44170
TDF477	POPTR_0014s17400	Pt-PSAL.2	Photosystem I reaction centre subunit XI	1.20E-169	JK475121	AT4G12800
TDF483	POPTR_0003s12150	---	BCL2-associated athanogene-like proteins	4.30E-90	JK475122	AT2G23150
TDF500	POPTR_0003s06560	Pt-LOX3.2	Lipoxygenase	2.30E-87	JK475123	AT3G03590

Aliases^a^ were obtained from the Joint Genome Institute (JGI: http://www.jgi.doe.gov/); Accession nos.^b^ were obtained from NCBI GeneBank (http://www.ncbi.nlm.nih.gov/genbank/); all sequences were also searched for at TAIR. The details are provided in the supplementary data (Additional file 6: Table S2).

### Quantitative real time RT-PCR analysis

**Figure 5:**
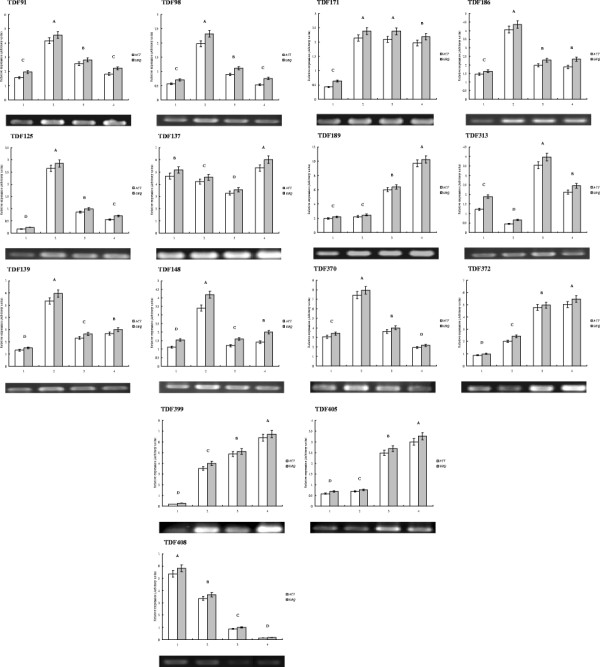
**Transcript of 15 selected *P. hopeiensis* genes at different stages of water-deficit treatment as determined by quantitative RT-PCR and semi-quantitative RT-PCR.** The Poplar *ACTINII-like* (accession number: EF145577) and *UBIQUITIN* genes were used as an internal controls. Genes expression patterns relative to the *ACTINII-like* gene were used as an internal control,and are represented as a white column (notated as *ACT*). Genes expression patterns relative to that *UBIQUITIN* gene were used as internal control, and are represented as a gray column (notated as *UBQ*). Significant differences between treatments (*P*≦0.01) are denoted by letters A-D, Bars indicate SE, 1–4, four time point of water-deficit treatment. Annotation of TDF91-408 were shown in Table 1.

Twenty-four TDFs representing genes with different expression patterns in response to water loss were subjected to quantitative real time RT-PCR (qPCR). The poplar *ACTINII-like* (accession: EF145577) and *UBIQUITIN* genes, which are constitutively expressed in performed with semi-quantitative real time RT-PCR analysis were selected as the internal control to which transcript abundance was normalized (Additional file 7: Figure S5). For most TDFs, transcript fold ratios were similar to the cDNA-AFLP results, indicating the reliability of that technique (Figure5). TDF125 and TDF189 displayed a higher fold-induction in the PCR analysis, which probably reflects the higher dynamic range of qPCR [23]. Meanwhile, these two TDFs were used to detect expression patterns in response to other stresses (Additional file 8: Figure S6). Only the expression pattern of TDF399 differed between the qPCR and cDNA-AFLP data. The expression of nine TDFs coding unknown proteins from different clusters was determined using real-time PCR (Additional file 9: Figure S7).

### Expressions of antioxidant and photosynthetic genes under drought stress

**Figure 6:**
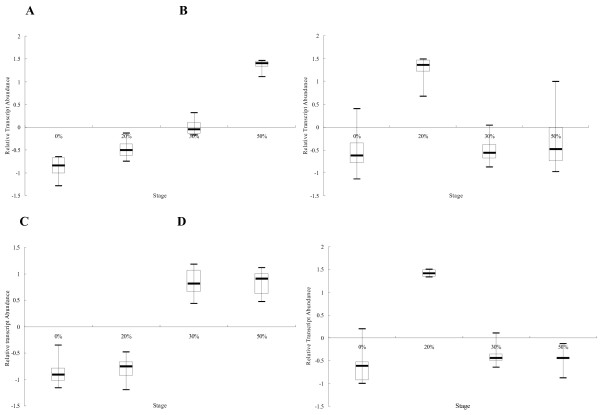
**Box plot illustrating the differential transcript pattern of antioxidant and photosynthetic genes under water stress.A**–**C**, Type I, II, and III expression patterns, respectively, of antioxidant genes in poplar under water loss treatment. Stage, four treatment time points (as in the following figure). **D**, photosynthetic genes transcript pattern in response to water stress.

We examined the expression patterns of genes associated with oxidative stress, which is related to the drought-stress response, and classified them into three types; Type I genes showed increased expression from 0% to 20% FWL, then a rapid decrease in expression from 20% to 30% FWL. In the last stage (50% FWL), most TDFs showed stable levels of expression, except for TDF440, which showed a slight increase (Figure6A). Annotation analyses showed that most of Type I genes were associated with cell membranes. Type II genes showed stable expression in the first and last stages of dehydration, but a significant increase in 30% FWL stage (Figure6B). Most of these genes were associated with the mitochondria. Type III genes showed increased expression throughout the drought treatment (Figure6C). GO analyses showed that most of these genes were associated with the mitochondria and the peroxisomes.

The expressions of many photosynthetic genes were significantly repressed during drought stress. We analyzed the expression of a number of genes encoding products associated with chlorophyll binding, Photosystem I (PSI), and PSII under drought stress. The expression of TDF50, which shows homology to the light-harvesting complex II protein LHCB1 (*LHCB1*), significantly decreased from 0% to 20% FWL, and then remained unchanged from 30% to 50% FWL. The expressions of TDF89, TDF139, TDF148, TDF370, and TDF477 showed a similar pattern, increasing markedly at the beginning of the drought treatment, peaking in the 20% FWL stage, decreasing dramatically from 20% to 30% FWL, and then remaining stable at 50% FWL (Figure6D).

### Stomatal responses to drought treatment

**Figure 7:**
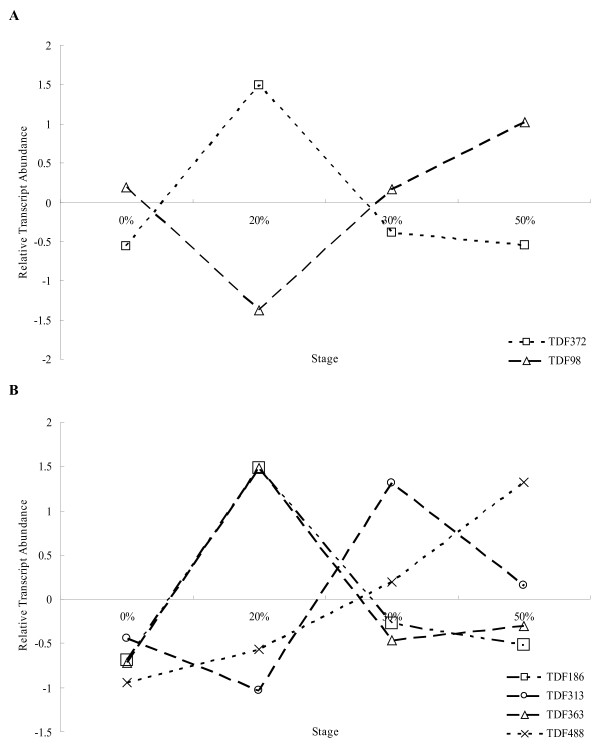
**Transcript patterns of genes related to stomatal regulation under drought stress.A**,TDF372 and TDF98 indicate the transcript pattern of K^+^ channel and Na^+^/H^+^ antiporter genes, respectively, in response to water stress. **B**, TDF186, TDF313, TDF363 and TDF488 indicate the transcript pattern of members of the *PP2C* gene family. Details of TDFs annotation are shown in Table 2.

To understand the stomatal responses to drought treatment at the molecular level, stomatal-related genes were selected from our cDNA-AFLP data and their expression patterns during drought stress were analyzed. Because potassium channels and Na^+^/H^+^ antiporters play an important roles in stomatal responses to environmental cues, we analyzed the expression of TDF98 and TDF372 for expression analyses. TDF98 shows homology to *Na*^*+*^*/H*^*+*^*EXCHANGER* (*NHX5*) gene*,* which encodes a plant NHX protein that has been described as an Na^+^/H^+^ antiporter [24]. Expression of TDF98 increased from the beginning of the drought treatment, peaked in the 20% FWL stage, and then decreased. Sequence comparisons indicated that TDF372 encodes a potassium transporter family protein. Expression of TDF372 decreased during the drought treatment, reached its lowest level in the 20% FWL stage, and then increased thereafter (Figure7A).

The protein phosphatase type C (PP2Cs) gene family plays a major role in stomatal responses and ABA signaling. We examined the expression profiles of four TDFs belonging to the PP2C family,and observed three different expression patterns: the expression of TDF186 and TDF363 increased from 0% to 20% FWL, then decreased thereafter. TDF313 exhibited the opposite expression pattern, decreasing from the 0% to 20% FWL stage, increasing from the 20% to 30% FWL, and then decreasing thereafter; and the expression of TDF488 was stable during the drought treatment. These different expression patterns suggest that the various members of the PP2C family might have different functions during drought stress (Figure7B).

### Expressions of genes related to cell expansion and apoptosis during drought stress

**Figure 8:**
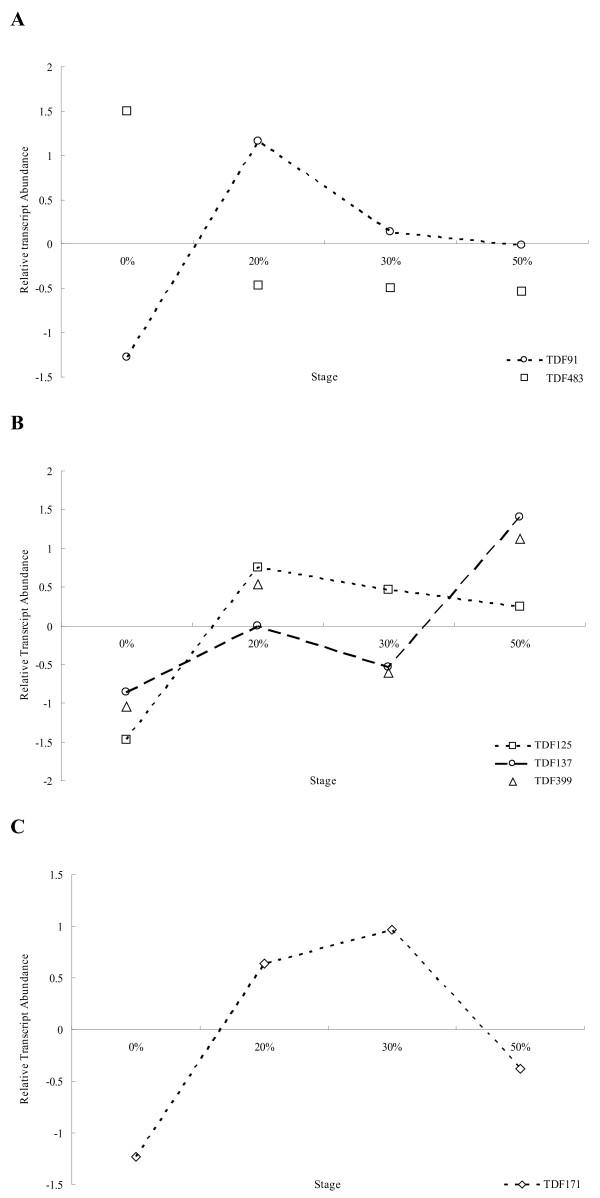
**Transcript patterns of genes that related to cell expansion, apoptosis and hormone signaling under water-deficit treatment.A**, TDF91 indicates the EXP4 gene transcript pattern in response to water stress, and TDF483 those of *ATHANO*-like genes. **B**, TDF125, TDF137 and TDF399 indicate transcript pattern of members of the ERD gene family. **C**, TDF171 indicate the *IAA14* genes transcript pattern in response to water stress. The details of TDF annotation are shown in Table 2.

Next, we analyzed the expression patterns of genes related to cell expansion and apoptosis during drought stress. TDF91 shows homology to *ALPHA-EXPANSIN 4*(*EXPA4*), which encodes alpha-expansin 4, a protein that increases the extensibility of the cell wall [25]. Expression of TDF91 increased from the 0% to 20% FWL stages, and then decreased until the 50% FWL stage (Figure8A). Meanwhile, BLAST results showed that TDF483 has homology to the *BCL2-ASSOCIATED ATHANOGENE-LIKE PROTEIN* (*BAG1*) gene, which encodes a BCL2-associated athanogene-like protein that restrains apoptosis. Expression of TDF483 was markedly decreased from the beginning of the drought treatment, reaching its lowest level in the 20% FWL stage and then remaining at a low level thereafter (Figure8A).

### Expressions of hormone-related genes during drought stress

Plant responses to different stresses are mediated by various hormonal signaling pathways. In the present study, four TDFs showed homology to genes associated with hormone signaling pathways. TDF125, TDF137, and TDF399 showed homology to *EARLY-RESPONSIVE TO DEHYDRATION 3* (*ERD3*), *ERD4,* and *ERD7*, respectively. These genes showed two types of expression pattern (Figure8B): expressions of TDF137 and TDF 399 increased from the beginning of the drought treatment until the 20% FWL stage, decreased to their lowest level in the 30% FWL stage, and then increased again. Whereas TDF125 increased sharply from the beginning of the drought treatment, reached a peak in the 20% FWL stage and then decreased thereafter. TDF171 shows homology to the *AUXIN-RESPONSIVE PROTEIN IAA 14*(*IAA14*) gene, which encodes an aux/IAA protein in the auxin-mediated signaling pathway. Expression of TDF171 gradually decreased in the 0% to 20% and 20% to 30% FWL stages, and moderately decreased in the 50% FWL stage (Figure8C).

## Discussion

### Drought transcriptome analysis

Most TDF sequences were annotated by sequence alignments using BLAST searches against NCBI databases. However, TDF45-1, TDF183, and TDF280-1 had no similar sequences in any of the databases. These sequences may thus represent genes that are unique to *P*. *hopeiensis*. TDF186, TDF313, TDF363, and TDF488 were annotated as different members of the *phosphatase 2 C* gene family, and showed different expression patterns (Figure7B). This result suggests that members of the *phosphatase 2 C* gene family have different and separate functions during the drought response. Transcriptome analysis showed some common drought-responsive processes. Functional analyses of the TDFs indicated that metabolism is the most important process during the drought response. The various metabolic genes showed different expression patterns, reflecting their different localizations within the cell. The expressions of nine non-annotated TDFs suggest the presence of putative novel genes that may be specific to poplar and might also play important roles in dehydration stress adaptation [26].

### Responses of photosynthesis and reactive oxygen species under drought stress

Previous studies have found that photosynthesis is usually unaffected by moderate drought in other species [27,28]. In poplar, full or partial maintenance of photosynthesis at moderate stress levels is accompanied by almost no transcriptional changes in photosynthesis genes before the most severe stress levels are attained [29]. However, in the present study, we found that some poplar genes related to photosynthesis were upregulated until the 20% FWL stage in poplar. This result definitely indicates that candidate photosynthesis genes are upregulated in response to water stress in poplar. The expression of *Arabidopsis* genes from PSI and PSII differ during moderate water-sterss treatment [1]. The fact that this was not the case in poplar suggests a different gene expression pattern in this species group.

Reactive oxygen species (ROS) are produced in different compartments of the plant cell under both stress and non-stress conditions [30]. High ROS concentrations are a symptom of stress. Plants must maintain the ROS within a certain level for normal cellular homeostasis. The ROS concentration in the cell is maintained by the antioxidant system, which comprises antioxidant molecules such as ascorbate, glutathione, and a-tocopherol, as well as antioxidant enzymes such as peroxidases, catalases, and dismutases [30,31]. The induction of members of the antioxidant system is correlated with stress severity. Under severe abiotic stress such as high light, salt stress, drought stress, and multiple concurrent stresses, antioxidant enzymes are differentially and strongly induced [32,33]. In the present research, the genes related to oxidative stress were divided into three types based on their expression patterns; Type I genes were rapidly upregulated at the onset of stress, indicating that they may be related to the early oxidative stress response. Type II genes showed increased expression from the 30% to the 50% of FWL stage, indicating that they are associated with the mid-phase of the stress response. The expression of type III genes gradually increased as the stress treatment continued, reaching a peak at the 50% FWL stage. Therefore, these genes may be involved in the final phase of the oxidative stress response.

### Stomata-related genes regulate the early response to drought stress

Stomatal closure under drought is an avoidance strategy adopted by plants to save water and which maintains turgor [34]. The channels and transporters responsible for ion transport across cell membranes are key regulators controlling stomatal aperture and water loss [35,36]. The *POTASSIUM TRANSPORTER PROTEIN* (*KT3*) gene and the *NA*^*+*^*/H*^*+*^*EXCHANGER* (*NHX5*) gene are down-or upregulated, consistent with their role in the inhibition of stomatal opening. The *KT3* gene encoding an inward K^+^ channel is expressed in guard cells. Under stress, the outward channels are induced and inward channels are repressed. Consistent with this, in the present study, the inward channel gene *KT3* showed the lowest expression level in the 20% FWL stage. The *NHX5* gene, which encodes an Na^+^/H^+^ antiporter that sequesters Na^+^ in vacuoles within plant cells, showed the highest expression level in the 20% FWL stage. Overexpression of *NHX5* promotes the accumulation of osmolytes (ions, soluble sugars, proline) to counter not only high salt high salt tolerance, but also high drought tolerance in *Broussonetia papyrifera*[24]. *KT3* exprssion gradually decreased as *NHX5* increased after the 20% FWL stage in *P. hopeiensis.* Changes in the Na^+^/K^+^ balance have been reported in *P. euphratica* in response to salt stress [37]. However, this is the first report of Na^+^/K^+^ balance regulation during drought stress in poplar; an Na^+^/K^+^ balance process may be involved in the poplar adaptive response to osmotic stress.

Under drought treatment, poplar initially shows a drastic decrease in leaf relative water content (LRWC) and stomatal conductance, but no change in the photosynthetic rate [29]. In the present study, the expression pattern of stomatal-related genes peaked during an early stage of drought stress. The PP2C genes are involved in ABA-signaling in the guard cells, and act as negative regulators of ABA signaling in stomata [38,39]. In the present research, four TDFs homologous to PP2Cs were induced at an early stage of drought treatment; this is consistent with the role of PP2Cs in mediating early responses. An early response in ABA signaling and regulation of guard cells under stress can improve tolerance to abiotic stress [40].

### Expression of genes related to cell expansion and apoptosis under drought stress

Cell expansion is a process of cell wall modification and loosening catalyzed by enzymatic and non-enzymatic protein components of the cell wall [41]. A previous transcriptome analysis of Arabidopsis under a progressive drought treatment showed the repression of many expansin genes [42], while in another study expansin genes were induced under mild osmotic stress [43]. In the present research, TDF91 (homologous to *EXPA4*) was dramatically upregulated at the beginning of drought stress, peaked in the 20% of FWL stage, and then decreased gradually. This early peak in expansin expression can be interpreted as acclimation to moderate drought by the cell wall adjustment. This is a common type of acclimation response, which can proceed by loosening and/or tightening of cell wall structure depending on the species, organ, and tissue [25]. However, whereas the expression of *EXPA4* was unchanged under progressive drought in *Arabidopsis*, we found that expression of TDF91 markedly decreased as the stress treatment continued. This result suggests that expansins may be regulated differently in different species. The plant cell wall is required not only for mechanical support, but also for growth and adaptation to adverse environmental conditions. Overexpression of expansin genes resulted in enhances growth in rice, and increases sensitivity to hormones and salt stress in *Arabidopsis*[44,45]. These observations suggest that expansins play a crucial role in the adaptive response of plants to abiotic stresses.

When proteins that have been induced by abiotic stress are damaged beyond repair, they must be eliminated to prevent aggregation. In this case, cells initiate the degradation of the damaged proteins or the destruction of entire cells (apoptosis) [46]. In *Arabidopsis*, members of the BAG protein family participate in several cellular processes, including apoptosis, proliferation, differentiation, and stress signaling [47]. In the present study, TDF483, which showed homology to the *BAG1* gene, exhibited decreased expression at the beginning of drought stress, indicating that this gene is sensitive to water loss. This finding suggests that it is important to maintain proteins in their functional conformations and prevent the aggregation of nonnative proteins under stress. Moreover, the expression of TDF483 decreased to its lowest point in the 20% FWL stage, and remained almost unchanged thereafter. This observation suggests that the 20% FWL stage is sufficient for *BAG* gene silencing, but not for apoptosis. Apoptotic regulation has been reported in foxtail millet (*Setaria italica* L.), and thus the role of this regulator in plants should be investigated at the cellular and molecular levels [48].

### Hormonal pathway responses to drought

Rapid adaptation to changing environmental conditions is essential for plant survival and for the development of tolerance to both abiotic and biotic stresses. Such tolerances can be achieved by distinct metabolic and physiological adjustments, which are mediated by a number of plant hormones, and are often specific to a certain type of stress [49]. As a central regulator of plants’ adaptation to environmental stress, ABA plays a crucial role in the regulation of transpirational water loss [50,51]. The early responsive to dehydration (*ERD*) gene is one of the key negative regulators of ABA responses in plants. Changes to the abundance of *ERD* transcript abundance modulate ABA responsiveness in *Arabidopsis*. In the present study, we demonstrated that the expression of *ERD3ERD4,* and *ERD7* responded apidly responded to water deficit. This implies that *ERD* genes are rapid drought-responsive genes, as are the *ERD* genes in Arabidopsis. The *ERD* gene family has at least 21 members. In the present study, *ERD3ERD4,* and *ERD7* showed different expression patterns, indicating that various members of the *ERD* gene family may have separate functions in the water stress response.

We observed changes in the IAA regulation pathway in response to drought. TDF171, an ortholog of *IAA14*, was upregulated at the beginning of the drought stress treatment. In poplar, RNAi-mediated downregulation of *IAA14* resulted in a severe dwarf phenotype with enhanced lateral shoot growth [52]. The upregulation of TDF171 observed in this study indicates that cell elongation was rapidly induced by moderate water stress. In general, adjustment of cell turgor pressure and elongation of root tip cells are positive responses to promote moisture absorption from the environment [53].

## Conclusions

Cell expansion in response to drought has been characterized in the maize (*Zea mays*) root system as an adaptation to low water potential [53]. Thus, in poplar, upregulation of cell extension-related genes in response to water deficit would enhance survival likelihood under water stress. Previous studies have not reported transcriptional changes in photosynthesis genes before the most severe stress levels [29]. However, in the present study, a series of candidate photosynthesis genes upregulated in response to water stress in poplar were found. This aids our understanding of the response of the photosynthesis to water stress. Transient responses to drought stress include stomatal closure, inactivation of K^+^ channels, and activation of Na^+^/H^+^ antiporters [1,54]. Changes in the expression of ABA-responsive genes and ROS scavengers, which are probably involved in signaling responses [55,56], were also detected in the present study. These abundant and diverse changes reflect the complexity of the drought response in plants.

We identified 415 differentially expressed TDFs using the cDNA-AFLP technique. The transcriptome profile showed that various biological processes were involved in drought stress, and a series of candidate genes were identified, including *ALDHLHCB1-3EXPAERDNHX5, KT3, BAG1IAA14*, and the *PP2C* gene or gene family. All of these genes were opening an interesting area for investigation of the responses of those genes to other stresses [57]. These data will facilitate the unraveling of the complex process of drought tolerance in poplar.

## Methods

### Plant materials

Explants from hydroponically grown *P*. *hopeiensis* shoots were sterilized and cultured on half-strength Murashige and Skoog (MS) medium supplemented with 0.5 mg·L^-1^ BA and 0.1 mg·L^-1^ NAA at 25 ± 1°C. Plants were grown under a 16 h photoperiod, with light provided by cool-white fluorescent lights (250 μmolm^-2^ s^-1^ PPFD). For rooting, regenerated shoots were subcultured on half-strength MS medium supplemented with 0.4 mg·L^-1^ IBA.

After 5–6 weeks of subculturing, the rooted plantlets were subjected to water deprivation. Their fresh weight was continuously monitored using a balance. Following the method of Pelah et al. [58], the plants were wilted at room temperature until they reached 80%, 70%, or 50% of their original fresh weight (corresponding to 20%, 30% and 50% FWL stages, respectively;the exact times at which plants reached those stages were at 35 min,1h10min,and 3h20min respectively), and then were kept in closed plastic bags for an additional 3 h. Rooted plantlets maintained in plastic bags for 3 h without prior wilting served as controls. Finally, 3–5 cultured rooted plantlets (>3 g) were sampled for each treatment, and were immediately frozen in liquid nitrogen and stored at −80°C until use.

### Total RNA extraction, mRNA isolation, and cDNA synthesis

Total RNA was extracted by the procedure of Chang et al. [59]. mRNA was isolated from total RNA (~0.5 mg for each treatment) using the PolyAtract mRNA Isolation System (Promega) (Additional file 10 and 11: Table S3 and Figure S8). Then double-stranded cDNA was then synthesized from mRNA with the Universal RiboClone cDNA Synthesis System (Promega) following the manufacturer’s protocol. The synthesized cDNA was dissolved in 50 μl TE buffer (10 mM Tris–HCl [pH 8.0], 1 mM EDTA).

### cDNA-AFLP analysis

cDNA-AFLP analyses were carried out using the AFLP Expression Analysis Kit (LI-COR), according to the manufacturer’s protocol. Approximately 100 ng cDNA from each treatment was digested with *Taq*I and *Mse*I restriction enzymes in a two-step reaction at 65°C and 37°C, respectively, for 2 h. After ligation of the adapter, 2.5 μl diluted (1:10) ligation mix was used as the cDNA template to perform pre-amplification, which was carried out in a 25 μl reaction mixture containing 10 μl pre-amp primer mix, 2.5 μl 10× amplification buffer, and 2 U Taq DNA polymerase (Invitrogen). The PCR thermal cycling conditions were as follows: 94°C for 5 min; 25 cycles of 94°C for 30 s, 56°C for 1 min, and 72°C for 1 min; and 72°C for 10 min, The amplified products were stored at 4°C. The obtained products were diluted 200-fold and then subjected to selective amplification. The reaction mixture contained 1.0 μl 10 × amplification buffer, 2 μl template, 2 μl *Mse*I primer containing dNTP, 0.5 μl IRDye™ 800-labeled *Taq*I primers, and 0.7 U Taq DNA polymerase (Invitrogen). The selective touch-down PCR conditions were as follows: 94°C for 5 min, 13 cycles of 94°C for 30 s, 65°C for 30 s (−0.7°C/cycle), and 72°C for 1 min, followed by 23 cycles of 94°C for 30 s, 56°C for 30 s, 72°C for 1 min, and 72°C for 10 min. Amplified products were then stored at 4°C. For selective amplification, 16 *Mse*I and 16 *Taq*I primers (256 primer combinations) were used in the reactions.

Each selected amplification product (10 μl) was mixed well with 5 μl loading buffer (Li-cor), denatured at 94°C for 3 min, and then chilled on ice before loading onto gels. The products were separated on 6% polyacrylamide gels, and electrophoresis and detection were performed using a two-dye automated DNA sequencer (model 4300, LI-COR). The electrophoretic running parameters were as follows: 1500 V, 40 mA, 40 W, 45°C, 150 min.

Gel images were quantitatively analyzed with Odyssey Application Software (version1.2), through which all visible AFLP fragments were scored and all individual band intensities were measured in each lane. The raw data were corrected for differences using a total lane intensity correction. To that end, the intensity values were summed per lane for each primer combination and each of the sums was divided by the maximal value to yield the correction factor. Finally, all raw data were divided by these correction factors. For the corrected data, the CV value (=SD/mean) of significant differences in expression genes was calculated. The higher the CV value, the higher the difference in transcript abundance levels [60]. The TDFs with CV values >0.5 were selected as significantly differentially expressed genes, and were subjected to cloning. For the selected TDFs, hierarchical clustering was performed with the Cluster and Treeview programs using a complete linkage algorithm and the Pearson’s correlation as a distance measure [61,62].

### TDF sequence analysis and gene ontology annotation

The bands corresponding to the selected TDFs were excised from gel and soaked in 30 μl TE buffer (10 mM Tris–HCl [pH 8.0], 1 mM EDTA) at 37°C for 3 h to elute the DNA. After centrifugation at 12000 rpm for 10 min, a 4 μl aliquot was used for re-amplification of the fragments in a 20 μl reaction mixture using the same primers as those used for pre-amplification. The PCR thermal cycling conditions were as follows: 30 s at 94°C, 30 s at 58°C, and 1 min at 72°C for 30 cycles. PCR products were characterized by separation on a 1.0% agarose gel, cloned into the pMD19-T vector (Takara), and sequenced.

The resulting TDF sequences were aligned against NCBI databases using BLASTX algorithm, with an *E*-value cut-off of 1.0E-5 to retrieve annotations. The remaining TDFs were aligned against *Populus* DB EST sequences (http://www.populus.db.umu.se/blast.php) and the *P*. *trichocarpa* genome sequence database (http://www.phytozome.net/search.php? show = blast&method = Org_Ptrichocarpa) using the BLASTX algorithm.

Nucleotide sequences were also analyzed using Blast2Go (Version 2.4.4) [63] for gene annotation, and to assign the DNA sequences to functional categories (e.g., biological process, molecular function, cellular component).

### Semi-quantitative RT-PCR

First-strand cDNA was synthesized from 1 μg total RNA using the reverse transcription system (Promega), according to the manufacturer’s instructions. The PCR reactions were performed using gene-specific primers with Takara Ex Taq (Takara, Dalian, China) in a final reaction volume of 20 μL, consisting of 13.25 μL ddH_2_O, 2.0 μL 10 × buffer, 2.0 μL 2.0 mmol·L^–1^ dNTP, 0.8 μL of each primer (at 10 μmol·L^–1^), 1 μL template, and 0.75 U Taq DNA polymerase. Amplifications were carried out using the following cycling parameters: preliminary denaturation (5 min, 94°C), then 25–30 cycles of denaturation (20 s, 94°C), annealing (20 s, 58°C) and extension (40 s, 72°C), and a final extension for 7 min at 72°C. Amplified products were stored at 4°C. PCR products were separated on a 1% agarose gel and visualized by ethidium bromide staining. The poplar *ACTINII*-like (GenBank accession number EF145577) and *UBIQUITIN* genes were selected as the internal controls. All primers are shown in Additional file 12: Table S4.

#### Real-time quantitative PCR verification

Quantitative PCR was performed using the TaKaRa ExTaq R PCR Kit and SyBR green dye (TaKaRa, Dalian, China) and a DNA Engine Opticon 2 machine (MJ Research). The PCR program included an initial denaturation at 94°C for 5 min; 40 cycles of 30 s at 94°C, 30s at 58°C and 30s at 72°C and a final melt-curve of 70–95°C. The generated melting curve was employed as a significant parameter to check the specificity of the amplified fragment. All reactions were triplicate for technical and biological repetitions of three plants, and the generated real-time data were analyzed using the Opticon Monitor Analysis Software ver.3.1 tool. For PCR using the primer pairs shown in Additional file 2: Table S2 were used. The efficiencies of the primer sets were calculated by performing real-time PCR on several dilutions of first-strand cDNAs. The efficiencies of the different primer sets were similar. The specificity of each primer set was checked by sequencing PCR products [64]. The results obtained for the different stages we analyzed were standardized to the levels of the *PtACTIN* gene and *Pt UBIQUITIN* genes.

### Data analysis

Scanned gel images were quantitatively analyzed using Odyssey Application Software version 1.2 (USA). All visible AFLP fragments were scored and all individual band intensities were measured. The intensity of bands was standardized according to the method of De Paepe and Breyne [60,62]. The coefficient of variation (CV) was calculated by dividing the standard deviation by the mean. The CV was used to establish a cut-off value, and expression profiles with a CV of ± 0.5 were considered to show significantly differential expression. The intensity of each band was variance-normalized using standard statistical methods as described by Breyne [62]. The Cluster and Tree View software packages were used for average linkage hierarchical clustering [61].

Real-time quantitative PCR data analysis using R software [65]. Means and stabdard error (SE) were calculated and compared using a one-way ANOVA. Differences in transcript abundance between treatments were determined using a Tukey’s HSD test.

#### Soluble sugars, protein and MDA measurements

Soluble sugar was measured as described by Mohsenzadeh et al. [66], and expressed as mg g^–1^ FW. Soluble protein contents were determined as described by Bradford [67], using bovine serum albumin as a standard. Soluble protein concentration was expressed as mg g^–1^ FW. Malondialdehyde (MDA) concentration was measured according to the method of Xu [68], and modified as follows: fresh leaves (0.3 g) were homogenized in 5 ml 5% trichloroacetic acid (TCA) solution. The absorbance of MDA was measured at 532, 600 and 450 nm. The MDA concentration was obtained using the following formula: C (μmol l^–1^) = 6.45 (A_532_ – A_600_) – 0.56A_450_. The MDA concentration was expressed as μmol g^–1^ FW.

## Abbreviations

FWL, Fresh weight loss; TDF, Transcript-derived fragments; PSI, Photosystem I; PSII, Photosystem II; ROS, Reactive oxygen species; LRWC, Leaf relative water content; CV, Coefficient of variation; ALDH, Aldehyde dehydrogenase; Bsp, Boiling stable protein gene; BAG1, BCL2-associated athanogene-like protein; DHN, Dehydrin gene; ERDs, Early-responsive to dehydration; EXPA4, Alpha-expansin 4; HSPs, Heat shock protein; IAA14, Auxin-responsive protein IAA14; KT3, Potassium transporter family protein; LHCB1, Light-harvesting complex II protein 1; LEAs, Late embryonic proteins; NHX5, Na+/H+ exchanger; PHOSPHATASE 2 C, Protein phosphatase type C.

## Misc

Yuepeng Song and Zeliang Wang, contributed equally to this work

Zhiyi Zhang is deceased.

## Competing interests

The authors declare that they have no competing interests.

## Authors’ contributions

YS, ZW, DZ, WB, and RR performed the research; YS, ZW, and DZ analyzed the data and prepared the manuscript; DZ and ZZ proposed the research project and guided the research. All authors read and approved the final manuscript.

## Supplementary Material

Additional file 1 **Figure S1. Physiological response to water-deficit stress in *P* . *hopeiensi* ( *P* **. ** *tomentosa* as a control).** A, Changes in soluble sugar content in response to water-deficit stressed. B, Changes in soluble protein content in response to water-deficit stress. C, Changes in malondialdehyde (MDA) content in response to water-deficit stress stage, four water-deficit stress time points. Click here for file

Additional file 2 Table S1. cDNA-AFLP primer information. Click here for file

Additional file 3 Figure S2. GO categorization of differentially expressed poplar genes during the 20% fresh weight loss. Click here for file

Additional file 4 Figure S3. GO categorization of differentially expressed poplar genes during the 30% fresh weight loss. Click here for file

Additional file 5 Figure S4. GO categorization of differentially expressed poplar genes during the 50% fresh weight loss stage. Click here for file

Additional file 6 Table S2. Transcript-derived fragments (TDFs) from P. hopeiensis showing drought-responsive expression, with homologies to genes in TAIR. Click here for file

Additional file 7 **Figure S5. Stable expression of poplar *ACTINII-like* (accession number: EF145577) and *UBIQUITIN* genes during the water stress used as the internal control.** Click here for file

Additional file 8 **Figure S6. TDF125 and 189 expression pattern response to stress.** A–E, representing cold, heat, H_2_O, NaCl, and ABA stress respectively.1-7, 0, 0.5, 1, 2, 5, 10, and 24 h time points after stress initiation. Click here for file

Additional file 9 Figure S7. Expression patterns of genes coding for unknown proteins. Click here for file

Additional file 10 Table S3. OD values of RNA samples. Click here for file

Additional file 11 Figure S8. Gel photos of RNA quality. Stage1-4, the four water treatment time points. S1-3, triplicate for biological repetitions. Click here for file

Additional file 12 Table S4. RT-PCR primer information. Click here for file
