# Supplementary material for: Transcriptional profiling by cDNA-AFLP analysis showed differential transcript abundance in response to water stress in Populus hopeiensis
Source: BMC Genomics. 2012 Jun 29;13:286. doi: 10.1186/1471-2164-13-286 (PMC3443059; doi:10.1186/1471-2164-13-286)
Supplement: Additional file 1 — Figure S1. Physiological response to water-deficit stress in P . hopeiensi ( P . tomentosa as a control). A, Changes in soluble sugar content in response to water-deficit stressed. B, Changes in soluble protein content in response to water-deficit stress. C, Changes in malondialdehyde (MDA) content in response to water-deficit stress stage, four water-deficit stress time points. [file 1471-2164-13-286-S1.doc]

**A**

Content of Soluble sugar (mg·g^-1^ FW)

**B**

Content of Soluble protein (mg·g^-1^ FW)

**C**

Content of malondialdehyde (MDA) (*u*mol·g^-1^ FW)

**Figure S1 Physiological response to water-deficit stress in *P*. *hopeiensi* (*P*. *tomentosa* as a control).** A, Changes in soluble sugar content in response to water-deficit stressed. B, Changes in soluble protein content in response to water-deficit stress. C, Changes in malondialdehyde (MDA) content in response to water-deficit stress Stage, four water-deficit stress time points.
