# Supplementary material for: Transcriptional profiling by cDNA-AFLP analysis showed differential transcript abundance in response to water stress in Populus hopeiensis
Source: BMC Genomics. 2012 Jun 29;13:286. doi: 10.1186/1471-2164-13-286 (PMC3443059; doi:10.1186/1471-2164-13-286)
Supplement: Additional file 2 — Table S1. cDNA-AFLP primer information. [file 1471-2164-13-286-S2.doc]

**Table S1 cDNA-AFLP primer information.**

| Primer No. | Primer sequences | Primer No. | Primer sequences |
| --- | --- | --- | --- |
| *Taq* I 1 | GATGAGTCCTGAGCGAA AC | *Mse* I 1 | GATGAGTCCTGAGTAAAC |
| *Taq* I 2 | GATGAGTCCTGAGCGAA AG | *Mse* I 2 | GATGAGTCCTGAGTAAAG |
| *Taq* I 3 | GATGAGTCCTGAGCGAA CA | *Mse* I 3 | GATGAGTCCTGAGTAACA |
| *Taq* I 4 | GATGAGTCCTGAGCGAA CT | *Mse* I 4 | GATGAGTCCTGAGTAACT |
| *Taq* I 5 | GATGAGTCCTGAGCGAA TG | *Mse* I 5 | GATGAGTCCTGAGTAAGA |
| *Taq* I 6 | GATGAGTCCTGAGCGAA TC | *Mse* I 6 | GATGAGTCCTGAGTAAGT |
| *Taq* I 7 | GATGAGTCCTGAGCGAA GT | *Mse* I 7 | GATGAGTCCTGAGTAATC |
| *Taq* I 8 | GATGAGTCCTGAGCGAA GA | *Mse* I 8 | GATGAGTCCTGAGTAATG |
| *Taq* I 9 | GATGAGTCCTGAGCGAA GC | *Mse* I 9 | GATGAGTCCTGAGTAAAA |
| *Taq* I 10 | GATGAGTCCTGAGCGAA GG | *Mse* I 10 | GATGAGTCCTGAGTAAAT |
| *Taq* I 11 | GATGAGTCCTGAGCGAA AT | *Mse* I 11 | GATGAGTCCTGAGTAACG |
| *Taq* I 12 | GATGAGTCCTGAGCGAA AA | *Mse* I 12 | GATGAGTCCTGAGTAACC |
| *Taq* I 13 | GATGAGTCCTGAGCGAA CG | *Mse* I 13 | GATGAGTCCTGAGTAAGC |
| *Taq* I 14 | GATGAGTCCTGAGCGAA CC | *Mse* I 14 | GATGAGTCCTGAGTAAGG |
| *Taq* I 15 | GATGAGTCCTGAGCGAA TA | *Mse* I 15 | GATGAGTCCTGAGTAATA |
| *Taq* I 16 | GATGAGTCCTGAGCGAA TT | *Mse* I 16 | GATGAGTCCTGAGTAATT |
