# Supplementary material for: Transcriptional profiling by cDNA-AFLP analysis showed differential transcript abundance in response to water stress in Populus hopeiensis
Source: BMC Genomics. 2012 Jun 29;13:286. doi: 10.1186/1471-2164-13-286 (PMC3443059; doi:10.1186/1471-2164-13-286)
Supplement: Additional file 3 — Figure S2. GO categorization of differentially expressed poplar genes during the 20% fresh weight loss. [file 1471-2164-13-286-S3.doc]

**Up**

**Down**

**Biological Process**

**Molecular Function**

**Cellular Component**

**Figure S2 GO categorization of differentially expressed poplar genes during the 20% fresh weight loss stage**
