# Supplementary material for: Transcriptional profiling by cDNA-AFLP analysis showed differential transcript abundance in response to water stress in Populus hopeiensis
Source: BMC Genomics. 2012 Jun 29;13:286. doi: 10.1186/1471-2164-13-286 (PMC3443059; doi:10.1186/1471-2164-13-286)
Supplement: Additional file 7 — Figure S5. Stable expression of poplar ACTINII-like (accession number: EF145577) and UBIQUITIN genes during the water stress used as the internal control. [file 1471-2164-13-286-S7.doc]

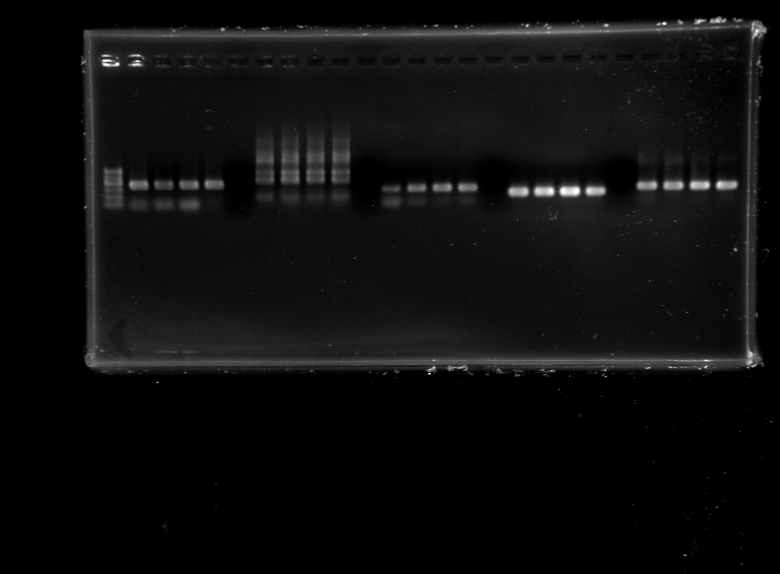


*ACTINII-like*


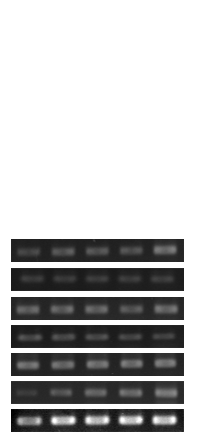


*ubiquitin* biquitin *-like*

**Figure S5 Stable expression of poplar *ACTINII-like* (accession number: EF145577)** **and *ubiquitin* genes during the water stress used as the internal control.**
