# Supplementary material for: Transcriptional profiling by cDNA-AFLP analysis showed differential transcript abundance in response to water stress in Populus hopeiensis
Source: BMC Genomics. 2012 Jun 29;13:286. doi: 10.1186/1471-2164-13-286 (PMC3443059; doi:10.1186/1471-2164-13-286)
Supplement: Additional file 8 — Figure S6. TDF125 and 189 expression pattern response to stress. A–E, representing cold, heat, H2O, NaCl, and ABA stress respectively.1-7, 0, 0.5, 1, 2, 5, 10, and 24 h time points after stress initiation. [file 1471-2164-13-286-S8.doc]

**A Cold**

1 2 3 4 5 6 7


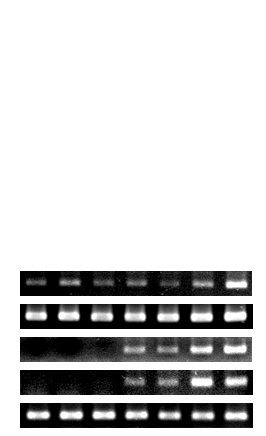


TDF125


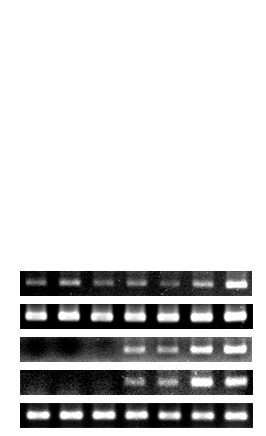


TDF189

**B Heat**

1 2 3 4 5 6 7


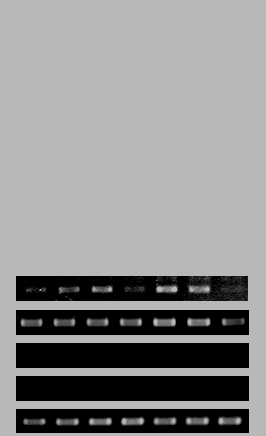


TDF125


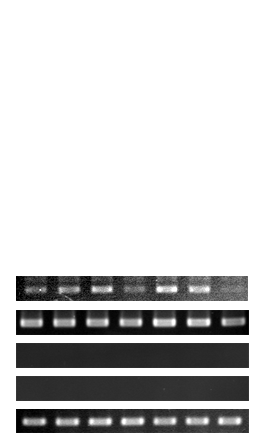


TDF189

**C H_2_O**

1 2 3 4 5 6 7


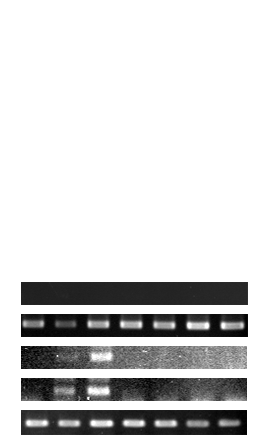


TDF125


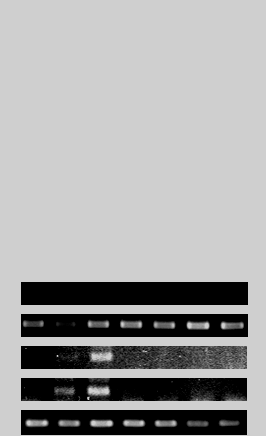


TDF189

**D Nacl**

1 2 3 4 5 6 7


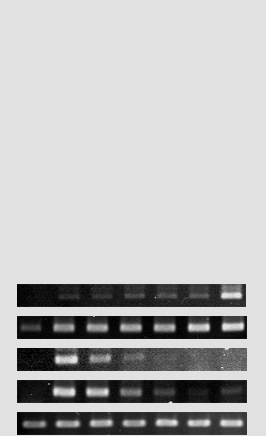


TDF125


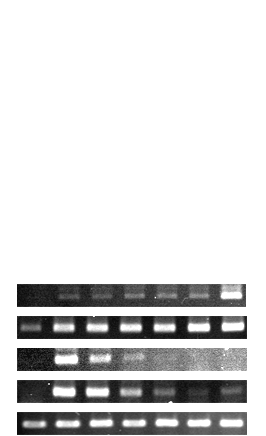


TDF189

**E ABA**

1 2 3 4 5 6 7


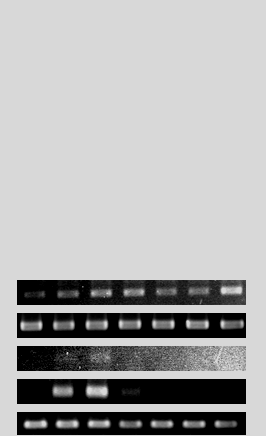


TDF125


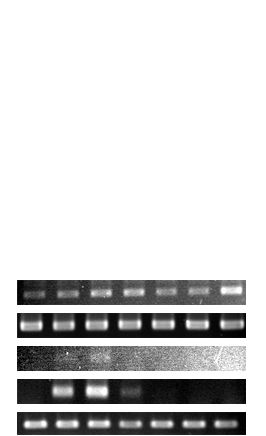


TDF189

**Figure S6 TDF125 and 189 expression pattern response to stress.** A–E, representing cold, heat, H_2_O, NaCl, and ABA stress, respectively. 1-7, 0, 0.5, 1, 2, 5, 10, and 24h time points after stress initiation.
