# Supplementary material for: Transcriptional profiling by cDNA-AFLP analysis showed differential transcript abundance in response to water stress in Populus hopeiensis
Source: BMC Genomics. 2012 Jun 29;13:286. doi: 10.1186/1471-2164-13-286 (PMC3443059; doi:10.1186/1471-2164-13-286)
Supplement: Additional file 9 — Figure S7. Expression patterns of genes coding for unknown proteins. [file 1471-2164-13-286-S9.doc]

**Clusters 1 TDF182 Clusters 2 TDF114**

**Clusters 5 TDF419 Clusters 6 TDF153**

**Clusters 7 TDF348 Clusters 8 TDF329**

**Clusters9 TDF444 Clusters11 TDF321**

**Clusters12 TDF355**

**Figure S7 Expression patterns of genes coding for unknown proteins.**
