# Supplementary material for: Transcriptional profiling by cDNA-AFLP analysis showed differential transcript abundance in response to water stress in Populus hopeiensis
Source: BMC Genomics. 2012 Jun 29;13:286. doi: 10.1186/1471-2164-13-286 (PMC3443059; doi:10.1186/1471-2164-13-286)
Supplement: Additional file 10 — Table S3. OD values of RNA samples. [file 1471-2164-13-286-S10.doc]

**Table S3. OD values of RNA samples**

| Stage | Sample | OD values | |
| --- | --- | --- | --- |
|  |  | OD260/OD230 | OD260/OD280 |
| 0% | 1 | 2.029 | 1.924 |
|  | 2 | 2.021 | 1.942 |
|  | 3 | 2.031 | 1.965 |
| 20% | 1 | 2.011 | 1.944 |
|  | 2 | 2.023 | 1.923 |
|  | 3 | 2.045 | 1.950 |
| 30% | 1 | 2.020 | 1.941 |
|  | 2 | 2.048 | 1.938 |
|  | 3 | 2.051 | 1.936 |
| 50% | 1 | 2.013 | 1.956 |
|  | 2 | 2.024 | 1.945 |
|  | 3 | 2.025 | 1.957 |
