# Supplementary material for: Transcriptional profiling by cDNA-AFLP analysis showed differential transcript abundance in response to water stress in Populus hopeiensis
Source: BMC Genomics. 2012 Jun 29;13:286. doi: 10.1186/1471-2164-13-286 (PMC3443059; doi:10.1186/1471-2164-13-286)
Supplement: Additional file 11 — Figure S8. Gel photos of RNA quality. Stage1-4, the four water treatment time points. S1-3, triplicate for biological repetitions. [file 1471-2164-13-286-S11.doc]

Stage 2

Stage 1

S3

S1

S2

S3

S2

S1


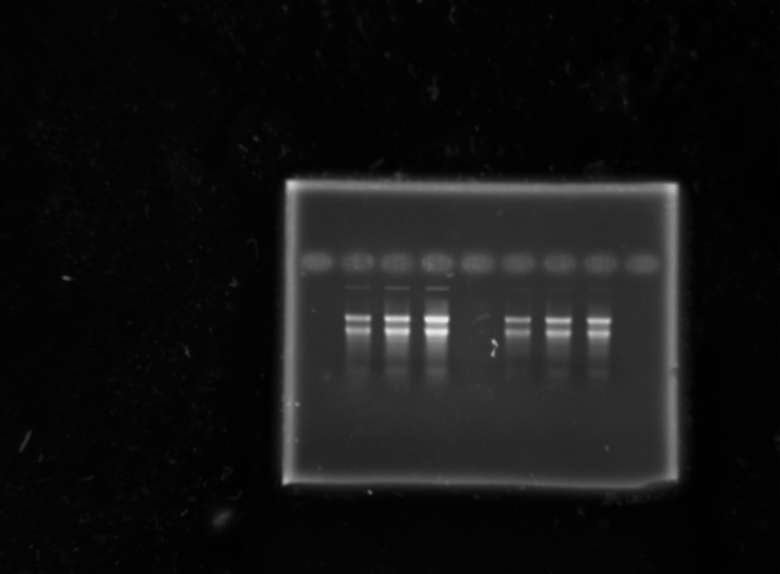


Stage 3

Stage 4

S3

S3

S2

S2

S1

S1


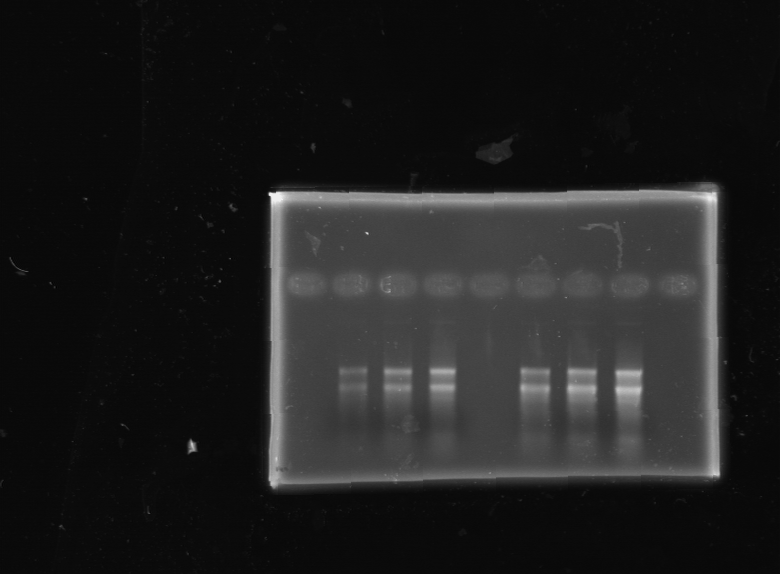


**Figure S8 Gel photos of RNA quality.** Stage1-4, the four water treatment time points. S1-3, triplicate for biological repetitions.
