# Supplementary material for: Transcriptional profiling by cDNA-AFLP analysis showed differential transcript abundance in response to water stress in Populus hopeiensis
Source: BMC Genomics. 2012 Jun 29;13:286. doi: 10.1186/1471-2164-13-286 (PMC3443059; doi:10.1186/1471-2164-13-286)
Supplement: Additional file 12 — Table S4. RT-PCR primer information. [file 1471-2164-13-286-S12.doc]

**Table S4 sqRT-PCR primer information.**

| Primer No. | Primer information | Product size | Efficiency of PCR reaction | | | Primer No. | Primer information | Product size | Efficiency of PCR reaction | | |
| --- | --- | --- | --- | --- | --- | --- | --- | --- | --- | --- | --- |
|  |  |  | S1 | S2 | S3 |  |  |  | S1 | S2 | S3 |
| TDF91 | F: TGACTGTGAACCTTATGCCTCC  R: CCCAGCCTGTCTTCCAAC | 105 bp | 98% | 97% | 99% | TDF321 | F: TACCGAGGGAAGGGAGCC  R: TCAGCCAACCTTCGCACA | 273bp | 98% | 101% | 98% |
| TDF98 | F: GTAGACTGCGTACCGACTG  R: TTTTGACGACGAAGAACAT | 140 bp | 97% | 100% | 98% | TDF329 | F: CCTCGTTTGGACTATGAA  R: CAGCCTCTTTGTCATCTC | 223 bp | 100% | 98% | 99% |
| TDF114 | F: AGTAACAGCCACAGAAGG  R: TACCGACTAAAGCATTGA | 356 bp | 102% | 99% | 99% | TDF348 | F: CCGAATGTATTTGCTGCT  R: GTCACTCGCCAAGTCCTC | 146 bp | 97% | 100% | 98% |
| TDF125 | F: ATGGCAGTGCGAATGGTT  R: TTCCTCTTTCCTGGTGGG | 74 bp | 100% | 100% | 99% | TDF355 | F: GATGAGTCCTGAGTAAGTG  R: TAGCCAATCAAACTACAG | 249 bp | 99% | 97% | 97% |
| TDF137 | F: GAACAGCAAGGAAAGTATCG  R: AGGAACCAGGCACTCAAA | 150 bp | 97% | 96% | 97% | TDF370 | F: AGCAATGGCGGAAGAGTT  R: CGGACAAAGGAGTTAGGGT | 264 bp | 99% | 98% | 97% |
| TDF139 | F: ATACCCATCTTCAAAGTCCA  R: TCTATCTATTAGCGGCTAGTGT | 79 bp | 100% | 98% | 98% | TDF372 | F: AGTCCTGAGTAAGGCGAAAT  R: TGCTAACATGCCACCAAA | 251 bp | 98% | 97% | 97% |
| TDF148 | F: CTCCCTTGAACCACCTCT  R: AATCCATCACCGACTACG | 273 bp | 94% | 96% | 95% | TDF399 | F: AGACTGCGTACCGAAAGG  R: TCCACATTAGGAGCCAAC | 189 bp | 96% | 98% | 97% |
| TDF153 | F: CTGAGTAATACCGCCAAG  R: TAGGACTGCGTACCGATG | 263 bp | 96% | 97% | 97% | TDF405 | F: AACTCCTGGACTGATAAATA  R: AGACTGCGTACCGAAAAT | 185 bp | 98% | 95% | 97% |
| TDF171 | F: GAAGGTGAGAAGGCAAGC  R: GCCAGGGCATCAGATAAT | 95 bp | 95% | 94% | 96% | TDF419 | F: ATAAATGAAGTAGTGGAGG  R: AAAGTCAAATGTTACGCT | 117 bp | 102% | 95% | 94% |
| TDF183 | F: TGAGTCCTGAGTAACGGG  R: AGATCCATCACGCATTCT | 147 bp | 100% | 97% | 99% | TDF444 | F: GATGAGTCCTGAGTAAAG  R: AATTCTGATTTCTTGAAG | 219 bp | 105% | 100% | 101% |
| TDF186 | F: TTGCTTTGATGGGGTCAT  R: ATTCGGTCGTTCTTGTGC | 71 bp | 103% | 99% | 100% | TDF483 | F: CCCATTACAGAAACACGAG  R: AGTAATCAAAAGTTTCCCATG | 135 bp | 98% | 100% | 98% |
| TDF189 | F: AATCAATGGCTCTTACGC  R: CCACCTTGTTTCTTCTTACTG | 240 bp | 101% | 100% | 100% | *ubiquitin* gene | F: CGTGGAGGAATGCAGATTTT  R: GATCTTGGCCTTCACGTTGT | 215 bp | 99% | 99% | 99% |
| TDF313 | F: CGTACCGAGGGCAAGTCT  R: TAATGGCAGTGGCAGGAT | 163 bp | 99% | 98% | 97% | *Actin-like* gene | F: GCCGAGCAGTATGAAGATCAAAG  R: CAGAAGCACTTCCTGTGGACGAT | 228 bp | 100% | 101% | 100% |
